# Genomic comparison of two *Streptococcus suis* serotype 1 strains recovered from porcine and human disease cases

**DOI:** 10.1038/s41598-023-32724-z

**Published:** 2023-04-03

**Authors:** Rujirat Hatrongjit, Nahuel Fittipaldi, Piroon Jenjaroenpun, Thidathip Wongsurawat, Suwattana Visetnan, Han Zheng, Marcelo Gottschalk, Anusak Kerdsin

**Affiliations:** 1 Department of General Sciences, Faculty of Science and Engineering, Kasetsart University Chalermphrakiat Sakon Nakhon Province Campus, Sakon Nakhon, 47000 Thailand; 2 GREMIP, Faculty of Veterinary Medicine, University of Montreal, Saint-Hyacinthe, QC Canada; 3 Division of Bioinformatics and Data Management for Research, Department of Research and Development, Faculty of Medicine Siriraj Hospital, Mahidol University, Bangkok, Thailand; 4 State Key Laboratory of Infectious Disease Prevention and Control, Collaborative Innovation Center for Diagnosis and Treatment of Infectious Diseases, Chinese Center for Disease Control and Prevention, National Institute for Communicable Disease Control and Prevention, Changping, Beijing, China; 5 Faculty of Public Health, Kasetsart University Chalermphrakiat Sakon Nakhon Province Campus, Sakon Nakhon, 47000 Thailand

**Keywords:** Microbiology, Microbial genetics

## Abstract

*Streptococcus suis* is a zoonotic pathogen that causes invasive infections in humans and pigs. Although *S. suis* serotype 2 strains are most prevalent worldwide, other serotypes are also occasionally detected. Herein, we investigated the genomes of two *S. suis* serotype 1 strains belonging to the clonal complex 1, which were recovered from a human patient and an asymptomatic pig, respectively. The genomes differed in pathotype, virulence-associated gene (VAG) profile, minimum core genome (MCG) typing, and antimicrobial resistance gene content. The porcine serotype 1 strain was sequence type (ST) 237 and MCG1, whereas the human serotype 1 strain was ST105 and MCG ungroupable. Both strains were susceptible to several antibiotics consisting of β-lactams, fluoroquinolones, and chloramphenicol. Resistance to tetracycline, macrolides, and clindamycin was observed, which was attributed to the genes *tet(O)* and *erm(B)*. Analysis of 99 VAG revealed *Hhly3*, *NisK, NisR, salK/salR, srtG, virB4*, and *virD4* were absent in both serotype 1. However, the porcine strain lacked *sadP* (Streptococcal adhesin P), whereas the human strain harbored *sadP1*. Phylogenetic analysis revealed that human *S. suis* ST105 strains from Vietnam were genetically the closest to the human serotype 1 strain, whereas porcine *S. suis* ST11 strains from China and Thailand were genetically the closest to the porcine strain.

## Introduction

*Streptococcus suis* causes invasive infections in swine^1^. Globally, serotypes 2, 1/2, 3, 4, 5 7, 8, 9, and 14 are the most frequently recovered from diseased pigs^1–3^. *S. suis* is also a zoonotic agent. Of late, especially in Southeast Asian countries, there has been a substantial increase in the number of human cases of *S. suis* occurring in patients who have reported close contact with one or more of infected pigs, contaminated pork-derived products, or consumption of raw pork products^4,5^. Among the 29 described serotypes of *S. suis*, serotype 2 is most associated with human infections^1,6^, although human disease due to serotypes 4, 5, 7, 9, 14, 16, 21, 24, and 31 has also been reported^1,7–11^. Notably, while *S. suis* serotype 1 has been commonly isolated from diseased pigs in Canada, Belgium, and the United States^1,2^, there is only one report (containing two cases) of serotype 1 human disease^12^ and notably, in the human report, assignment of strains to serotype 1 was based on biochemical tests only and not confirmed using antisera or polymerase chain reaction (PCR).

Herein, we sequenced the genome of a *S. suis* strain isolated from a true serotype 1 human disease case. By comparing the genomic traits of this strain to a porcine serotype 1 genome, we studied novel insights into the genomic characteristics, putative virulence genes, the prediction of the pathogenic capacity, and the antimicrobial gene repertoire of this important *S. suis* serotype.

## Results and discussion

### Identification of *S. suis* serotype 1

**Figure 1:**
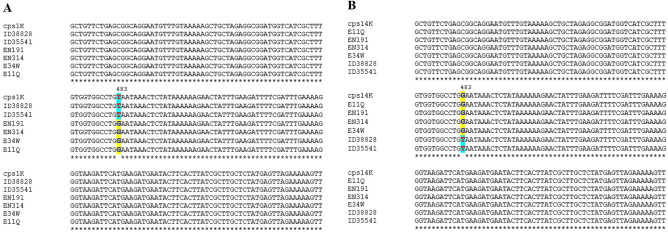
Alignment of *cps1K* (**A**) and *cps14K* (**B**) at the position of 483 to identify serotype 1 or 14 among the strains ID38828, ID35541, E11Q, E34W, EN191, and EN314. The alignment result indicated ID38828 and ID35541 were the serotype 1 and E11Q, E34W, EN191, and EN314 were the serotype 14.

Using a novel multiplex PCR test^13^, we first retyped the 46 strains in our collection previously identified using multiplex PCR^14^ as serotype 1 or 14. Among them, 2 strains were confirmed as serotype 1 by the new multiplex PCR^13^, PCR–RFLP of the *cpsK* locus^15^ and SNP at position 483 of *cpsK*^16^ (Fig. 1). The first strain was ID38828, isolated in 2011 from the blood of a human patient with septicemia in a hospital in Eastern Thailand. The second serotype strain (ID35541) was isolated from the tonsils of a clinically healthy pig in 2010 in Northern Thailand.

### General genomic information

The completed genomes of the two *S. suis* serotype 1 were 2,074,728 bp and 2,097,918 bp for strains ID38828 and ID35541, respectively. Strain no. ID38828 contained 1,963 coding sequences (CDS) and 12 rRNA and 56 tRNA genes. ID35541 had 1,987 CDS and 12 rRNA and 56 tRNA genes. No plasmids were detected in either strain using PlasmidFinder and PLACNETw.

### Antimicrobial resistance

Globally available antimicrobial resistance data for *S. suis* showed that *S. suis* strains recovered from both humans and pigs have high resistance to tetracycline and moderate-to-high resistance to macrolides, such as erythromycin^17–24^. We identified resistance to tetracycline, erythromycin, azithromycin, and clindamycin in the two serotype 1 strains, which, on the other hand, were susceptible to penicillin, ceftriaxone, levofloxacin, and chloramphenicol.

ResFinder 4.1 identified the genes *tetO* and *ermB* which confer resistance to tetracycline and macrolide-lincosamide-streptrogramin (MLS_B_), respectively, in the two serotype 1 strains. Two aminoglycoside-resistance genes (*ant(6)-Ia* and *aph(3')-III*) were additionally detected in the porcine strain ID35541. The prevalence and number of antimicrobial resistance genes have been shown to be variable among the different serotypes^25^. In addition, several studies have shown that the genes *tet(O)* and *erm(B)* are widely observed among pig and human S. suis isolates of various serotypes worldwide^18,25–29^. The genes *ant(6)-Ia* and *aph(3')-III* have been reported in several *S. suis* strains isolated from pigs in Canada, China, Korea, and Thailand^17,25,28,30^. Three existing aminoglycoside-modifying enzyme types have been described in *S. suis*, consisting of aminoglycoside N-acetyltransferases encoded by *aac* genes, aminoglycoside O-phosphotransferases encoded by *aph* genes, and aminoglycoside O-nucleotidyl transferases encoded by *ant* genes^18^.

**Figure 2:**
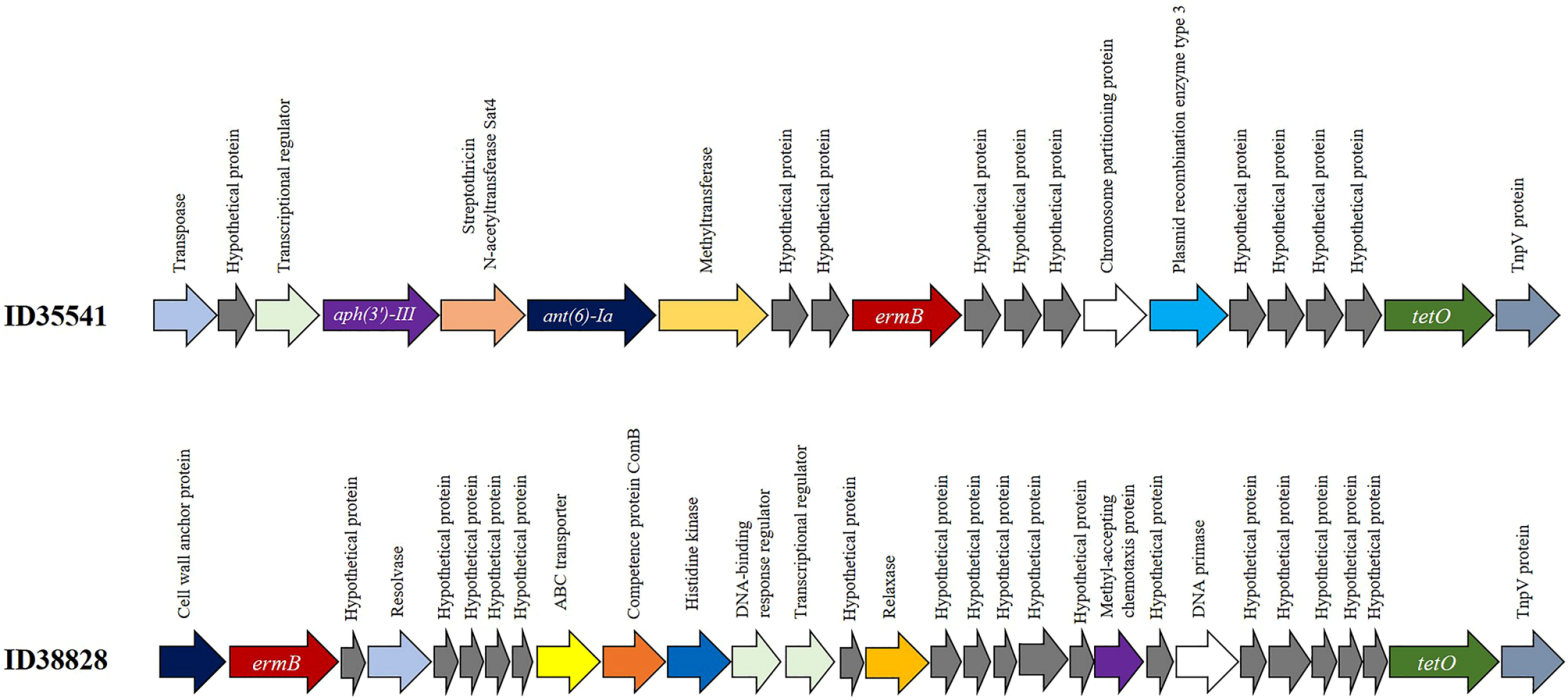
Genetic organization of antimicrobial resistant genes in two *Streptococcus suis* serotype 1 strains.

As shown in Fig. 2, the organization of the *tetO* and *ermB* genes was different in the strains ID35541 and ID38828. ID35541 had the genes *ant(6)-Ia* and *aph(3')-III* upstream of *ermB* and *tetO*; the genetic organization of these antimicrobial resistance genes was flanked by transposase genes.

### Virulence-associated genes

Two other studies have described the presence of genes encoding a copper-exporting ATPase 1, a type I restriction-modification system S protein, two hypothetical proteins (*SSU_RS03100* and *SSU_RS09155*), and an RNA-binding protein that could be useful to define a *S. suis* pathogenic pathotype, while a gene encoding a putative sugar ATP-binding cassette transporter could be a marker of strains possessing a non-pathogenic pathotype^31,32^. Our two serotype 1 strains had all pathogenic pathotype marker genes and lacked the non-pathogenic pathotype marker gene (the putative sugar ATP-binding cassette transporter). This may suggest that both the serotype 1 strains under investigation belonged to the pathogenic pathotype (diseased-associated). Note that three pathotype markers (copper-exporting ATPase 1, type I restriction-modification system S protein and putative sugar ATP-binding cassette transporter) were evaluated on small *S. suis* strains collected from England and Wales region^31^, whereas *SSU_RS03100* and, *SSU_RS09155* and the RNA-binding protein gene were evaluated with only North American isolates^32^. Large *S. suis* strains with difference of regions or countries, isolation sources, serotypes and STs should be further evaluated for the useful of this pathotyping system.

The two serotype 1 genomes under investigation were screened for the presence of 99 VAGs^33,34^. Among these 99 VAGs, 20 were considering to be putative zoonotic virulence factors (PZVF; Table 1)^34^. Eight VAGs from 99 VAGs (*Hhly3*, *NisK, NisR, salK, salR, srtG, virB4*, and *virD4*) were absent from both the ID38828 and ID35541 genomes (Table 1). Among 20 PZVF, only *Hhly3*, *NisK*, and *NisR* were absent in both strain (Table 1). In addition, Streptococcal adhesin P (*sadP*), one of PZVF, was absent from the genome of the porcine strain ID35541. Whereas *sadP1* was observed in the human strain ID38828. This *sadP1* was widely distributed and correlating with the CC1^35^, according to our serotype 1 strain which belonged in CC1. Both the serotype 1 strains in this study had a classical VAG profile (*epf/sly/mrp*), suggesting the potential for increased virulence. However, the swine strain ID35541 present a large variant of *epf* and a small variant of *mrp*, its classical VAG profile was epf ^*^/sly^+^*/mrp*^*S*^. Whereas, the human strain ID38828 showed no variants of *epf* and *mrp*, the profile was *epf*^+^*/sly*^+^*/mrp*^+^.

**Table 1:** Distribution of 99 virulence-associated genes in *Streptococcus suis* serotype 1.

No.	Virulence-associated genes	ID38828	ID35541	Potential zoonotic virulent factor
1	cbp40omp40	** + **	** + **	Yes
2	Fhb-1	** + **	** + **	Yes
3	Fhb-2 (Streptococcal adhesin P or sadP)	+	−	Yes
4	hylA	+	+	Yes
5	Hhly3	−	−	Yes
6	IdeS	+	+	Yes
7	IgA protease (*zmp*)	+	+	Yes
8	IgdE	+	+	Yes
9	mrp	+	+ ^*$*^	Yes
10	neuB	+	+	Yes
11	NisK	−	−	Yes
12	NisR	−	−	Yes
13	pnuC	+	+	Yes
14	rfeA (RTX toxin)	+	+	Yes
15	rgg	+	+	Yes
16	sly	+	+	Yes
17	SP1	+	+	Yes
18	*sbp1* (srtBCD cluster)	+	+	Yes
19	*sbp2* (srtBCD cluster)	+	+	Yes
20	tran	+	+	Yes
21	1910HK	+	+	No
22	1910HR	+	+	No
23	6-phosphogluconate dehydrogenase	+	+	No
24	103-adhesion protein	+	+	No
25	Abpb	+	+	No
26	AdcR	+	+	No
27	Amylopullulanase	+	+	No
28	Arginine deiminase	+	+	No
29	Autolysin	+	+	No
30	CcpA	+	+	No
31	cdd	+	+	No
32	ciaHR	+	+	No
33	Collagenase	+	+	No
34	CovR	+	+	No
35	Dipeptidylpeptidase IV	+	+	No
36	dltA	+	+	No
37	Dpr	+	+	No
38	Endo-*b*-N-acetylglucosaminase D	+	+	No
39	Enolase	+	+	No
40	epf	+	+ ^#^	No
41	FbpS	+	+	No
42	FeoB	+	+	No
43	Fur	+	+	No
44	GAPDH	+	+	No
45	gh92	+	+	No
46	Glutamate dehydrogenase	+	+	No
47	Glutamine synthetase	+	+	No
48	gpmA-38 KDa protein	+	+	No
49	gtfA	+	+	No
50	guaAB	+	+	No
51	HP0245	+	+	No
52	HtpS	+	+	No
53	ihk	+	+	No
54	irr	+	+	No
55	Lgt	+	+	No
56	lmb	+	+	No
57	lspA lipoprotein	+	+	No
58	LuxS	+	+	No
59	manN	+	+	No
60	Mannose specific EIIAB	+	+	No
61	nadR	+	+	No
62	oppA	+	+	No
63	OFS-serum opacity factor	+	+	No
64	Peptidase-SSU0458	+	+	No
65	Permease-SSU0501	+	+	No
66	Permease-SSU0835	+	+	No
67	pgdA	+	+	No
68	prsA	+	+	No
69	purA	+	+	No
70	purD	+	+	No
71	revS	+	+	No
72	salK/salR	−	−	No
73	Surface antigen one (sao)	+	+	No
74	scrB	+	+	No
75	scrR	+	+	No
76	serS	+	+	No
77	sntA	+	+	No
78	sodA	+	+	No
79	Spy-M3-0908	+	+	No
80	srtA	+	+	No
81	srtF	+	+	No
82	srtG	−	−	No
83	ssa	+	+	No
84	ssadS	+	+	No
85	ssnA-nuclease	+	+	No
86	sspA	+	+	No
87	SSU05-0473	+	+	No
88	SSU05-1311	+	+	No
89	SMU61-like (SSU05-0053)	+	+	No
90	ssPep	+	+	No
91	Stp	+	+	No
92	treR	+	+	No
93	Trigger factor	+	+	No
94	TroA	+	+	No
95	virA	+	+	No
96	virB4	−	−	No
97	virD4-TraG	−	−	No
98	yzpA	+	+	No
99	zur	+	+	No

^#^Was a large variant epf (epf*).

^*$*^Was a small variant mrp^s^.

### Genomic comparison

**Figure 3:**
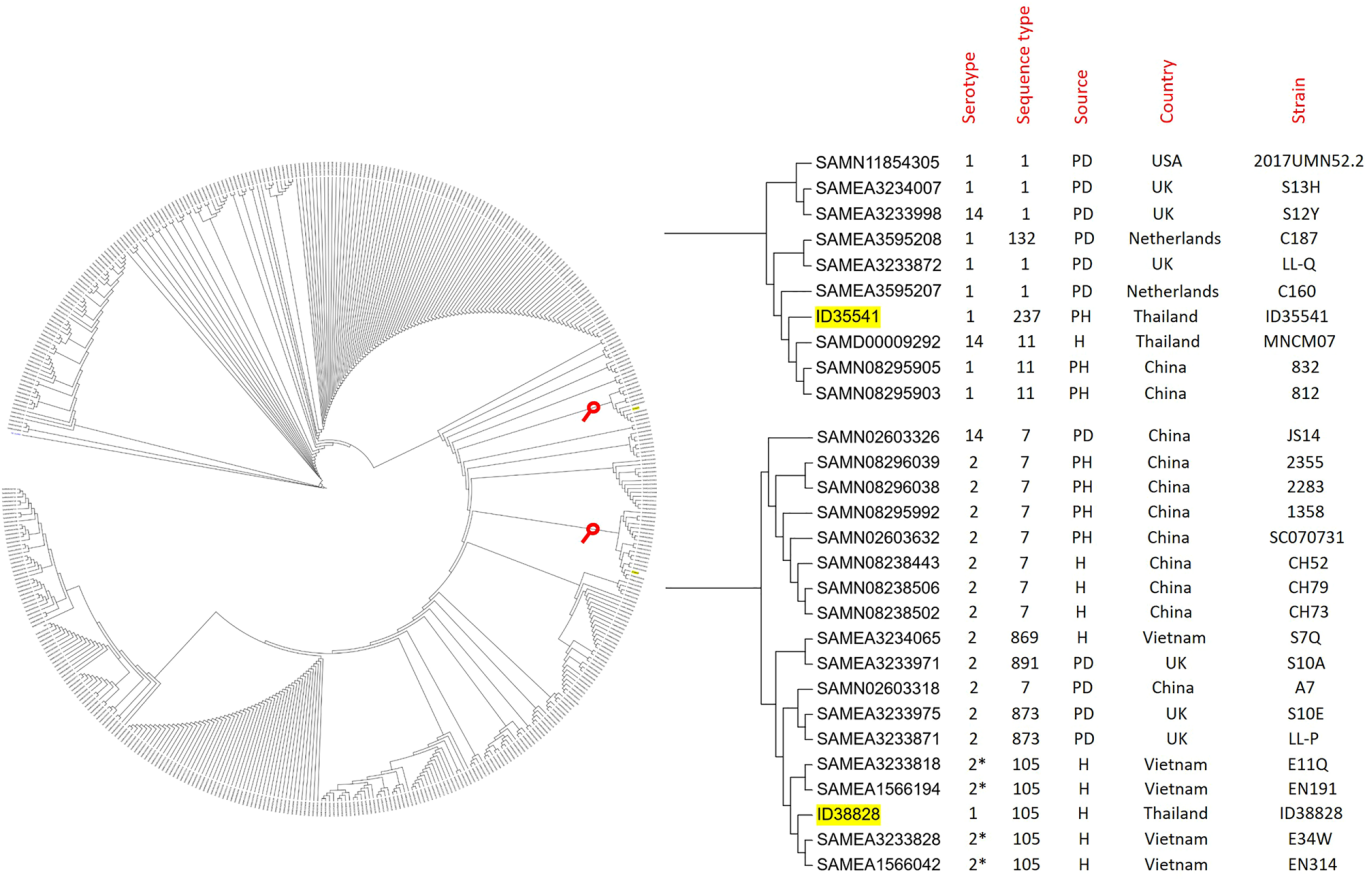
A phylogenetic tree based on the core genome SNPs of 430 genomic sequences of *S. suis* clonal complex 1 was constructed using the maximum likelihood method by FastTree v2.1.10 tool and visualized using interactive Tree of Life tool. The whole genome sequence of *S suis* serotype 1 strains in the current study are highlighted in yellow. * These original serotype 2 strains assigned in BioSample of GenBank were retyped as serotype 14 in this study.

MLST analysis assigned strain ID38828 to ST105 and strain ID35541 to ST237. Both ST105 and ST237 are included in CC1. We analyzed 428 CC1 strains download from GenBank together with our two strains. As shown in Fig. 3, the porcine serotype 1 strain ID35541 (ST237) was very closely related to the ST11 strains MNCM07 (Thailand), 812 and 832 (both from China), and ST1 strain C160 from The Netherlands. The human serotype 1 strain ID38828 (ST105) clustered with the Vietnamese strains EN314, E34W, EN191, and E11Q. These ST105 Vietnamese strains are serotype 2 (retyped as serotype 14 in this study) isolates recovered from human infections https://www.ncbi.nlm.nih.gov/biosample/SAMEA1566042; https://www.ncbi.nlm.nih.gov/biosample/SAMEA3233828; https://www.ncbi.nlm.nih.gov/biosample/SAMEA1566194; https://www.ncbi.nlm.nih.gov/biosample/SAMEA3233818. *S. suis* ST105 strains have previously been reported almost exclusively in Southeast Asian countries, especially Thailand and Vietnam^5,36,37^, https://pubmlst.org/bigsdb?order=id&designation_field1=s_1_ST&designation_value1=105&set_id=0&submit=1&designation_operator1==&page=query&db=pubmlst_ssuis_isolates, accessed on Nov 18, 2022). It appears that ST105 is an endemic strain in this region. However, ST105 strains of serotype 1 have also been documented in pigs in the UK (https://pubmlst.org/bigsdb?order=id&designation_field1=s_1_ST&designation_value1=105&set_id=0&submit=1&designation_operator1==&page=query&db=pubmlst_ssuis_isolates; accessed on Nov 18, 2022).

Analysis of the MCG group showed that the ST237 porcine isolate ID35541 was MCG group 1. This group includes the virulent CC1 strains (especially ST1 and ST7) associated with human infections, death, and outbreaks^19^. MCG group 1 also contains a higher number of virulent genes^19^. However, the human ST105 isolate ID38828 was ungroupable by MCG analysis, as were the Vietnamese ST105 strains (EN314, E34W, EN191, and E11Q) most closely related to ID38828. The MCG ungroupable cluster also contained diverse *S. suis* serotypes and STs, which collectively had a higher number of virulence genes than strains belonging to MCG groups 2–7^19^. It could be that this group was also virulent because some human-infected strains fell in this group.

**Figure 4:**
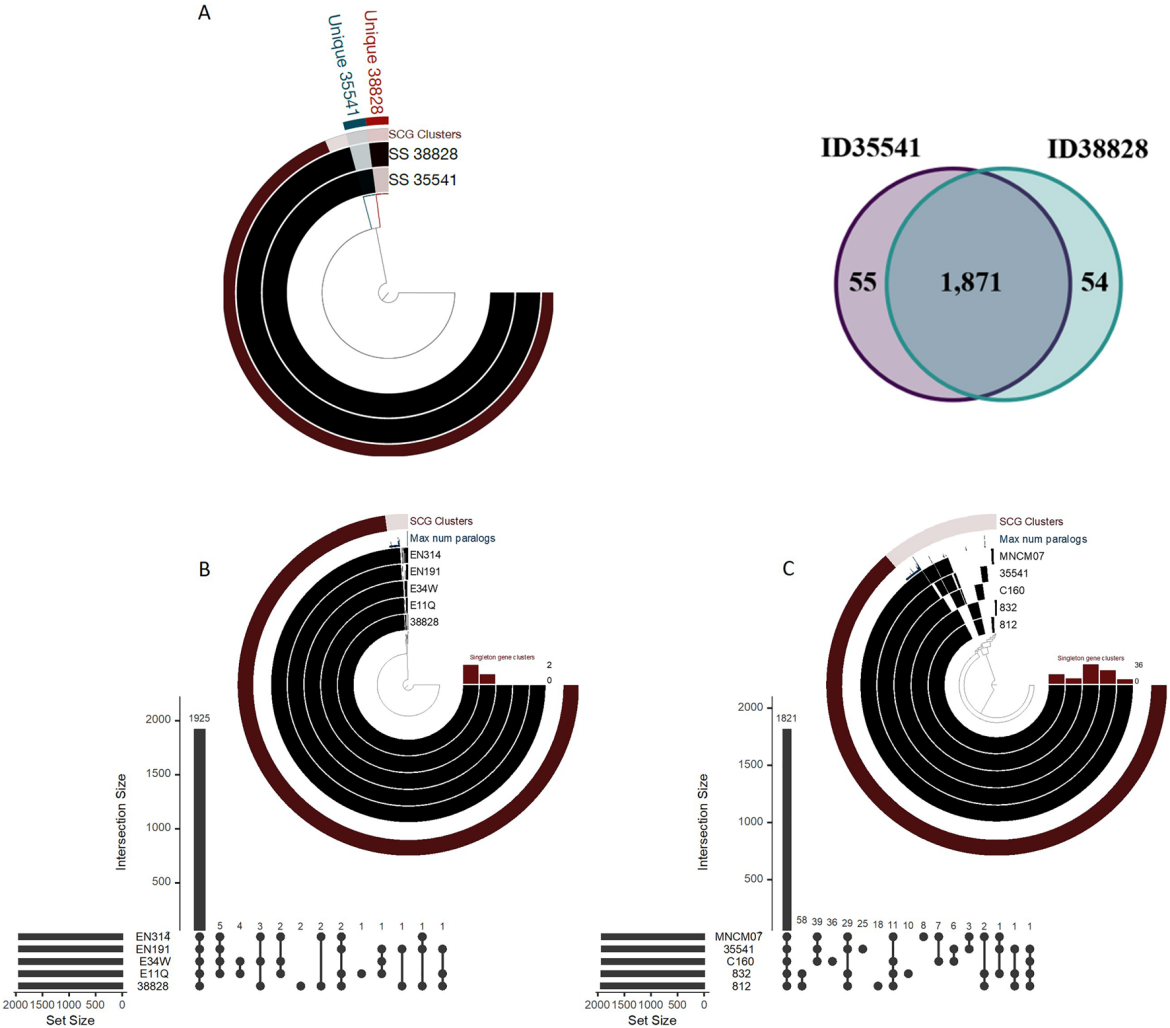
Pangenome analysis representation based on anvi'o software. **(A)** Pangenome reconstructed with two complete genomes of *S. suis* strains ID35541 and ID38828. Each ring in the graph represents an individual *S. suis* genome and each ray corresponds with a given gene homolog. The two inner layers are colored in black to mark gene clusters found in that genome or left with a translucent background if the gene cluster is absent from that genome. The third layer shows density plot of number of paralog genes. The outermost ring illustrates the single-copy genes (SCG; brown color) and accessary genes of strain ID35541 and ID38828 in torque and magenta highlight, respectively. Based on anvi’o pangenome analysis, there are 1871 core genes common between two strains ID38828 and ID35541. The accessory genes of strains ID38828 and ID35541 show 54 and 55 genes, respectively. **(B)** Pan-genome reconstructed with 5 genomes of *S. suis* strains ID38828, EN314, EN191, E34W, and E11Q. **(C)** Pan-genome reconstructed with 5 genomes of *S. suis* strains ID35541, MNCM07, C160, 832, and 812. Each ring in the graph (**B** and **C**) represents an individual *S. suis* genome and each ray corresponds to a given gene homolog. The five inner layers are in black to designate gene clusters in that genome or translucent if the gene cluster is absent. The third layer shows a density plot of number of paralog genes. The outermost ring illustrates the single-copy genes (SCG; brown). UpsetR showing the number of genes that are shared and unique between the genomes.

Pangenome analysis of the two *S. suis* serotype 1 strains and their closest relatives is presented in Fig. 4. Both our strains showed difference in 54 and 55 unique genes for strains ID38828 (human) and ID35541 (porcine), respectively. Interestingly, the human-derived strains ID38828, E11Q, EN191, E34W, and EN314 were highly homologous, with only 2 and 1 unique genes present in strains ID38828 and E11Q, respectively, whereas no unique gene found in the strains EN191, E34W, and EN314 (Table S1 and Fig. 4). Two unique genes of strain ID38828 encoded hypothetical proteins for both, while one unique gene of E11Q was similar to membrane protein. Comparison of our porcine strains ID35541 with strains MNCM07, C160, 812, and 832 were relatively more distantly related, with 25, 8, 36, 18, and 10 unique genes (Fig. 4 and Table S2) for ID35541, MNCM07, C160, 812, and 832, respectively.

It is interested that the genomic comparison among the human ST105 strains revealed no capsule genes different, actually our strain ID38828 and the Vietnamese strains (E11Q, EN314, EN191, and E34W) were different serotype as mentioned above. This may suggest that they are same serotype. To clarify this hypothesis, we determined capsule specific genes (*cpsI* and *cpsJ*) of the strains E11Q, EN191, E34W, and EN314. The specific capsule genes of these four strains showed positive only *cps1I*, *cps1J*, *cps14I*, and *cps14J*, whereas *cps2I*, *cps2J, cps1/2I*, and *cps1/2J* were not found for in all Vietnamese strains. It indicated that these four Vietnamese strains were either serotype 1 or 14, but not serotype 2 as mention in the GenBank data. Analysis of SNP at position 483 in *cps1K* and *cps14K* to identify serotype 1 or 14 revealed that all four strains were serotype 14 (Fig. 1).

Although the human strains were of different serotypes (serotype 1 for ID38828 and serotype 14 for EN314, E34W, EN191, and E11Q), genetically, they both were ST105. This suggested that they may represent an event of capsule switching because capsular locus of serotypes 1 and 14 are highly similar along capsule locus^38,39^. Capsular switching has been documented among serotype 2 strains^40,41^. Therefore it highly seems to occur between serotypes 1 and 14. In addition, the serotype 14-ST105 were also found and circulated in humans in Thailand^36,42^. It may be possible that Thai ST105 isolate of serotype 1 (also serotype 14) and the Vietnamese serotype 14 strain share a common ancestor circulating in the region as demonstrated in the Fig. 3.

## Conclusion

Comparison of the genome sequences of two *S. suis* serotype 1 strains (one human and one porcine) and the closest relatives strains revealed very few regions of difference and relatively important similarity. Based on the schematic systems of pathotyping and the VAG profile used for classifying the *S. suis* serotype 2 strains, the two serotype 1 strains investigated here are bona fide members of virulent groups. These strains belong to MCG-1 and MCG ungroupable for ST237 (ID35541) and ST105 (ID38828), respectively. They carry both the *tet*(O) and *ermB* genes conferring resistance to tetracycline, macrolide, and lincosamide; in addition, the porcine isolate ID35541 had aminoglycoside resistance genes (*ant(6)-Ia* and *aph(3')-III*). Resistance to tetracycline, erythromycin, azithromycin, and clindamycin were observed in the two serotype 1 strains, whereas, both were susceptible to penicillin, ceftriaxone, levofloxacin, and chloramphenicol.

## Materials and methods

### Bacterial strain re-typing and antimicrobial susceptibility

We used a recently described improved multiplex PCR, capable of discriminating between serotypes 1 and 14^13^, to re-type a total of 46 *S. suis* strains present in our collection; these had been assigned to serotype 14 or 1. In total, two serotype 1 strains were identified from these 46 strains. One strain (strain no. ID38828) was from a human patient, while the second was of porcine origin (strain no. ID35541). Confirmation of serotype 1 assignment was performed based on PCR–RFLP as described elsewhere^15^.

The broth microdilution technique was used according to the standards defined in the M100 (32nd edition) guidelines of the Clinical and Laboratory Standard Institute (CLSI) to determine the minimum inhibitory concentrations (MICs) of penicillin and ceftriaxone^43^. Susceptibilities to other antimicrobials (azithromycin, erythromycin, tetracycline, clindamycin, levofloxacin, and chloramphenicol) were determined using the disk diffusion technique following the 2022 CLSI-M100 guidelines^43^. Since there are currently no breakpoints recommended for *S. suis*, those for the viridans group streptococci were used, as defined in the guidelines^43^. The *Streptococcus pneumoniae* ATCC49619 strain was used for quality control purposes.

### Whole-genome sequencing

Bacterial genomic DNA samples extracted using ZymoBIOMICS DNA Kits (Zymo Research, CA, USA) were sequenced using the Oxford Nanopore Technologies (ONT) and Illumina platforms as described previously^44^. Hybrid assemblies with the ONT and Illumina data were performed using Unicycler v0.4.8^45^ and the genome sequences were checked for quality using QUAST v5.0.2^46^. Genome sequences were submitted to the NCBI Prokaryotic Genome Annotation Pipeline (PGAP v4.12) for annotation. The default parameters were used for all software unless otherwise specified.

### Bioinformatics analysis

The serotype of the strains was further confirmed by the occurrence of a single-nucleotide substitution at the position 483 of the *cpsK* locus^16^ differentiating reference *S. suis* serotypes 1 and 14 (GenBank accession no. JF273644 and AB737822, respectively) using the Center for Genomic Epidemiology’s MyDbFinder 2.0. Sequence types (ST) were identified by comparing the sequences against the PubMLST database (https://pubmlst.org/organisms/streptococcus-suis). Minimum core genome (MCG) sequence typing was performed according to the procedures described elsewhere^19^. We screened the genomes of the serotype 1 strains using MyDbFinder 2.0 for the presence of up to 99 genes previously described to be virulence-associated genes (VAG) (Table 1)^33,34^.

Antimicrobial resistance genes were detected using ResFinder 4.1^47^. Plasmid replicons were analyzed using PlasmidFinder 2.1 and PLACNETw^48,49^. Pangenome analyses were performed with the anvi’o v7 workflow^50^. This workflow identified gene clusters and single-copy genes in study genomes, including ID38828, ID35541, EN314, EN191, E34W, E11Q, MNCM07, C160, 832, and 812. All genomes, in fasta format, were submitted to pangenome analysis using the 'anvi-run-workflow' script. Genes were annotated using anvi-run-ncbi-cogs. All genomes were added to a new anvi'o genomes storage using the 'anvi-gen-genomes-storage' application. Then, the program ‘anvi-pan-genome’ ran pan-genomic analysis on all the stored genomes using NCBI's blastp tool. We used 'anvi-import-misc-data' to import additional metadata and 'anvi-compute-genome-similarity' to compute the average nucleotide identity (ANI) using the pyANI tool (https://github.com/widdowquinn/pyani). The pangenome was visualized in anvi'o using the 'anvi-display-pan' application. The whole pangenome was divided into core and accessory bins based on gene cluster frequency.

### Phylogenetic analysis

Totally, 430 CC1 genomes were included in phylogenetic analysis. *S. pneumoniae* genome ATCC700669 (accession no. NC_011900)^51^ was used as out-group to root the tree. Single-nucleotide polymorphisms (SNPs) were detected using MUMmer v3.23, and the genome sequence of *S. suis* strain P1/7 (accession no. NC_01292) was used as a reference^52^. A phylogenetic tree based on the core genome SNPs was constructed using the maximum likelihood method by FastTree v2.1.10 described in a previous study^19^. Bootstraps were performed with 1,000 replicates. The phylogenetic tree was visualized using the iTOL V4 software^53^.

### Accession number

The genome sequences of the two *S. suis* serotype 1 strains were deposited in the NCBI GenBank under Bioproject accession number PRJNA691075 for strain numbers ID38828 and ID35541, respectively.

### Ethics statement

Ethical review and approval were not required because no human specimens or data were used in the current study.

## Supplementary Information

Supplementary Table S1.

Supplementary Table S2.

## Data Availability

The assembled genomic sequences in the current study were deposited under the BioProject PRJNA691075 with accession number CP109941 (ID38828) and CP109942 (ID35541).

## References

[1] Goyette-Desjardins G, Auger JP, Xu J, Segura M, Gottschalk M (2014). *Streptococcus suis*, an important pig pathogen and emerging zoonotic agent-an update on the worldwide distribution based on serotyping and sequence typing. Emerg. Microbes. Infect..

[2] Lacouture S, Olivera YR, Mariela S, Gottschalk M (2022). Distribution and characterization of *Streptococcus suis* serotypes isolated from January 2015 to June 2020 from diseased pigs in Québec, Canada. Can. J. Vet. Res..

[3] Prüfer TL (2019). Molecular typing of *Streptococcus suis* strains isolated from diseased and healthy pigs between 1996–2016. PLoS ONE.

[4] Segura M (2020). *Streptococcus suis* research: Progress and challenges. Pathogens.

[5] Kerdsin A, Segura M, Fittipaldi N, Gottschalk M (2022). Sociocultural factors influencing human *Streptococcus suis* disease in Southeast Asia. Foods.

[6] Okura M (2016). Current taxonomical situation of *Streptococcus suis*. Pathogens.

[7] Callejo R (2014). Atypical *Streptococcus suis* in man, Argentina, 2013. Emerg. Infect. Dis..

[8] Hatrongjit R (2015). First human case report of sepsis due to infection with *Streptococcus suis* serotype 31 in Thailand. BMC Infect. Dis..

[9] Kerdsin A (2017). Emergence of *Streptococcus suis* serotype 9 infection in humans. J. Microbiol. Immunol. Infect..

[10] Liang P (2021). Genomic and pathogenic investigations of *Streptococcus suis* serotype 7 population derived from a human patient and pigs. Emerg. Microbes. Infect..

[11] Nghia HD (2008). Human case of *Streptococcus suis* serotype 16 infection. Emerg. Infect. Dis..

[12] Kopić J, Paradzik MT, Pandak N (2002). *Streptococcus suis* infection as a cause of severe illness: 2 cases from Croatia. Scand. J. Infect. Dis..

[13] Thu IS (2021). L, Direct detection of *Streptococcus suis* from cerebrospinal fluid, positive hemoculture, and simultaneous differentiation of serotypes 1, 1/2, 2, and 14 within single reaction. Pathogens.

[14] Kerdsin A (2014). *Streptococcus suis* serotyping by a new multiplex PCR. J. Med. Microbiol..

[15] Matiasovic J (2020). Resolution of *Streptococcus suis* serotypes 1/2 versus 2 and 1 versus 14 by PCR-restriction fragment length polymorphism method. J. Clin. Microbiol..

[16] Athey TB (2016). Determining *Streptococcus suis* serotype from short-read whole-genome sequencing data. BMC Microbiol..

[17] Aradanas M, Poljak Z, Fittipaldi N, Ricker N, Farzan A (2021). Serotypes, virulence-associated factors, and antimicrobial resistance of *Streptococcus suis* isolates recovered from sick and healthy pigs determined by whole-genome sequencing. Front. Vet. Sci..

[18] Dechêne-Tempier M (2021). Update on the mechanisms of antibiotic resistance and the mobile resistome in the emerging zoonotic pathogen *Streptococcus suis*. Microorganisms.

[19] Chen C (2013). Minimum core genome sequence typing of bacterial pathogens: A unified approach for clinical and public health microbiology. J. Clin. Microbiol..

[20] Hernandez-Garcia J (2017). Patterns of antimicrobial resistance in *Streptococcus suis* isolates from pigs with or without streptococcal disease in England between 2009 and 2014. Vet. Microbiol..

[21] Hoa NT (2011). The antimicrobial resistance patterns and associated determinants in *Streptococcus suis* isolated from humans in southern Vietnam, 1997–2008. BMC Infect. Dis..

[22] Ichikawa T, Oshima M, Yamagishi J, Muramatsu C, Asai T (2020). Changes in antimicrobial resistance phenotypes and genotypes in *Streptococcus suis* strains isolated from pigs in the Tokai area of Japan. J. Vet. Med. Sci..

[23] Matajira CEC (2020). *Streptococcus suis* in Brazil: Genotypic, virulence, and resistance profiling of strains isolated from pigs between 2001 and 2016. Pathogens.

[24] Yongkiettrakul S (2019). Antimicrobial susceptibility of *Streptococcus suis* isolated from diseased pigs, asymptomatic pigs, and human patients in Thailand. BMC Vet. Res..

[25] Ma L (2021). Genomic insight into the antimicrobial resistance of *Streptococcus suis* - six countries, 2011–2019. China CDC Wkly..

[26] Bojarska A (2016). *Streptococcus suis* in invasive human infections in Poland: Clonality and determinants of virulence and antimicrobial resistance. Eur. J. Clin. Microbiol. Infect. Dis..

[27] Chen L (2013). Antimicrobial susceptibility, tetracycline and erythromycin resistance genes, and multilocus sequence typing of *Streptococcus suis* strains from diseased pigs in China. J. Vet. Med. Sci..

[28] Yongkiettrakul S (2021). Genome sequences of antibiotic-resistant *Streptococcus suis* strains isolated from human patients and diseased and asymptomatic pigs in Thailand. Infect. Genet. Evol..

[29] Zhang C (2020). Capsular serotypes, antimicrobial susceptibility, and the presence of transferable oxazolidinone resistance genes in *Streptococcus suis* isolated from healthy pigs in China. Vet. Microbiol..

[30] Gurung M (2015). Molecular basis of resistance to selected antimicrobial agents in the emerging zoonotic pathogen *Streptococcus suis*. J. Clin. Microbiol..

[31] Wileman TM (2019). Pathotyping the zoonotic pathogen *Streptococcus suis*: Novel genetic markers to differentiate invasive disease-associated isolates from non-disease-associated isolates from England and Wales. J. Clin. Microbiol..

[32] Estrada AA, Gottschalk M, Gebhart CJ, Marthaler DG (2022). Comparative analysis of *Streptococcus suis* genomes identifies novel candidate virulence-associated genes in North American isolates. Vet. Res..

[33] Willemse N (2016). An emerging zoonotic clone in the Netherlands provides clues to virulence and zoonotic potential of *Streptococcus suis*. Sci. Rep..

[34] Roodsant TJ, Van Der Putten BCL, Tamminga SM, Schultsz C, Van Der Ark KCH (2021). Identification of *Streptococcus suis* putative zoonotic virulence factors: A systematic review and genomic meta-analysis. Virulence.

[35] Ferrando ML (2017). Streptococcal Adhesin P (SadP) contributes to *Streptococcus suis* adhesion to the human intestinal epithelium. PLoS ONE.

[36] Kerdsin A (2018). Genotypic diversity of *Streptococcus suis* strains isolated from humans in Thailand. Eur. J. Clin. Microbiol. Infect. Dis..

[37] Mai NT (2008). *Streptococcus suis* meningitis in adults in Vietnam. Clin. Infect. Dis..

[38] Okura M (2013). Genetic analysis of capsular polysaccharide synthesis gene clusters from all serotypes of *Streptococcus suis*: Potential mechanisms for generation of capsular variation. Appl. Environ. Microbiol..

[39] Wang K, Fan W, Cai L, Huang B, Lu C (2011). Genetic analysis of the capsular polysaccharide synthesis locus in 15 *Streptococcus suis* serotypes. FEMS Microbiol. Lett..

[40] Zhu Y (2021). Comparative genetic analyses provide clues about capsule switching in *Streptococcus suis* 2 strains with different virulence levels and genetic backgrounds. Microbiol. Res..

[41] Okura M (2021). Capsular polysaccharide switching in *Streptococcus suis* modulates host cell interactions and virulence. Sci. Rep..

[42] Kerdsin A (2009). Clonal dissemination of human isolates of *Streptococcus suis* serotype 14 in Thailand. J. Med. Microbiol..

[43] Clinical and Laboratory Standards Institute (CLSI). Performance standards for antimicrobial susceptibility testing–32nd Edition. M100. Wayne, PA. (2022).

[44] Kerdsin A (2021). Genomic characterization of *Streptococcus suis* serotype 24 clonal complex 221/234 from human patients. Front. Microbiol..

[45] Wick RR, Judd LM, Gorrie CL, Holt KE (2017). Unicycler: Resolving bacterial genome assemblies from short and long sequencing reads. PLoS Comput. Biol..

[46] Gurevich A, Saveliev V, Vyahhi N, Tesler G (2013). QUAST: Quality assessment tool for genome assemblies. Bioinformatics.

[47] Bortolaia V (2020). ResFinder 4.0 for predictions of phenotypes from genotypes. J. Antimicrob. Chemother..

[48] Carattoli A (2014). In silico detection and typing of plasmids using PlasmidFinder and plasmid multilocus sequence typing. Antimicrob. Agents. Chemother..

[49] Vielva L, de Toro M, Lanza VF, de la Cruz F (2017). PLACNETw: A web-based tool for plasmid reconstruction from bacterial genomes. Bioinformatics.

[50] Eren AM (2015). Anvi'o: An advanced analysis and visualization platform for 'omics data. PeerJ.

[51] Croucher NJ (2009). Role of conjugative elements in the evolution of the multidrug-resistant pandemic clone *Streptococcus pneumoniae*^Spain23F^ ST81. J. Bacteriol..

[52] Holden MT (2009). Rapid evolution of virulence and drug resistance in the emerging zoonotic pathogen *Streptococcus suis*. PLoS ONE.

[53] Letunic I, Bork P (2019). Interactive Tree of Life (iTOL) v4: Recent updates and new developments. Nucleic Acids Res..

